# Supplementary material for: Intersectional, anterograde transsynaptic targeting of neurons receiving monosynaptic inputs from two upstream regions
Source: Commun Biol. 2022 Feb 21;5:149. doi: 10.1038/s42003-022-03096-3 (PMC8860993; doi:10.1038/s42003-022-03096-3)
Supplement: Supplementary file 2 — Description of Additional Supplementary Files [file 42003_2022_3096_MOESM2_ESM.pdf]

Description of Additional Supplementary Files

File name: Supplementary Data 1
Description: Source data underlying graphs.
