# Supplementary material for: Intersectional, anterograde transsynaptic targeting of neurons receiving monosynaptic inputs from two upstream regions
Source: Commun Biol. 2022 Feb 21;5:149. doi: 10.1038/s42003-022-03096-3 (PMC8860993; doi:10.1038/s42003-022-03096-3)
Supplement: Supplementary file 4 — Reporting Summary [file 42003_2022_3096_MOESM4_ESM.pdf]

nature portfolio

Corresponding author(s): Takuma Kitanishi, Kenji Mizuseki

Last updated by author(s): January 10, 2022

Reporting Summary

Nature Portfolio wishes to improve the reproducibility of the work that we publish. This form provides structure for consistency and transparency in reporting. For further information on Nature Portfolio policies, see our Editorial Policies and the Editorial Policy Checklist.

Statistics

For all statistical analyses, confirm that the following items are present in the figure legend, table legend, main text, or Methods section.

n/a	Confirmed	
☐	☒	The exact sample size (n) for each experimental group/condition, given as a discrete number and unit of measurement
☐	☒	A statement on whether measurements were taken from distinct samples or whether the same sample was measured repeatedly
☐	☒	The statistical test(s) used AND whether they are one- or two-sided<br>*Only common tests should be described solely by name; describe more complex techniques in the Methods section.*
☒	☐	A description of all covariates tested
☒	☐	A description of any assumptions or corrections, such as tests of normality and adjustment for multiple comparisons
☐	☒	A full description of the statistical parameters including central tendency (e.g. means) or other basic estimates (e.g. regression coefficient) AND variation (e.g. standard deviation) or associated estimates of uncertainty (e.g. confidence intervals)
☐	☒	For null hypothesis testing, the test statistic (e.g. F, t, r) with confidence intervals, effect sizes, degrees of freedom and P value noted<br>*Give P values as exact values whenever suitable.*
☒	☐	For Bayesian analysis, information on the choice of priors and Markov chain Monte Carlo settings
☒	☐	For hierarchical and complex designs, identification of the appropriate level for tests and full reporting of outcomes
☒	☐	Estimates of effect sizes (e.g. Cohen's d, Pearson's r), indicating how they were calculated

Our web collection on statistics for biologists contains articles on many of the points above.

Software and code

Policy information about availability of computer code

Data collection	Confocal images were acquired using ZEN (ver. 2012, SP1, black edition and ver. 2.3, blue edition).
Data analysis	Cells were counted with Image-based Tool for Counting Nuclei (Center for Bio-image Informatics at University of California, Santa Barbara) and Fiji (ver. 2.1.0). Data were summarized with Excel (ver. 2019, Microsoft) and MATLAB (ver. R2017b, Mathworks). Statistical analysis was performed with SPSS Statistics (ver. 21, IBM).

For manuscripts utilizing custom algorithms or software that are central to the research but not yet described in published literature, software must be made available to editors and reviewers. We strongly encourage code deposition in a community repository (e.g. GitHub). See the Nature Portfolio guidelines for submitting code & software for further information.

Data

Policy information about availability of data

All manuscripts must include a data availability statement. This statement should provide the following information, where applicable:

- Accession codes, unique identifiers, or web links for publicly available datasets
- A description of any restrictions on data availability
- For clinical datasets or third party data, please ensure that the statement adheres to our policy

The pAAV-hSyn-Flpo-3×FLAG plasmid is available from Addgene (no. 173047). The data supporting this study are available from the corresponding authors upon reasonable request.

Field-specific reporting

Please select the one below that is the best fit for your research. If you are not sure, read the appropriate sections before making your selection.

☒ Life sciences ☐ Behavioural & social sciences ☐ Ecological, evolutionary & environmental sciences

For a reference copy of the document with all sections, see nature.com/documents/nr-reporting-summary-flat.pdf

Life sciences study design

All studies must disclose on these points even when the disclosure is negative.

Sample size	Sample sizes were chosen according to previously published studies and experience.
Data exclusions	AAV-misinjected mice with no Cre or Flpo expression in the presynaptic regions were excluded from the analysis.
Replication	All AAV injections were replicated using two or more mice and the results were consistent across individual animals.
Randomization	Randomization was not performed. We allocated all groups of mice to be compared in single batches of AAV injections.
Blinding	Cell counting was performed without the knowledge of the experimental group.

Reporting for specific materials, systems and methods

We require information from authors about some types of materials, experimental systems and methods used in many studies. Here, indicate whether each material, system or method listed is relevant to your study. If you are not sure if a list item applies to your research, read the appropriate section before selecting a response.

Materials & experimental systems

n/a	Involved in the study	
☐	☒	Antibodies
☐	☒	Eukaryotic cell lines
☒	☐	Palaeontology and archaeology
☐	☒	Animals and other organisms
☒	☐	Human research participants
☒	☐	Clinical data
☒	☐	Dual use research of concern

Methods

n/a	Involved in the study	
☒	☐	ChIP-seq
☒	☐	Flow cytometry
☒	☐	MRI-based neuroimaging

Antibodies

Antibodies used	Mouse anti-Cre recombinase (cat. MAB3120, clone 2D8, lot 2776878, Millipore)<br>Rabbit anti-FLAG (DYKDDDDK) (cat. RFLG-45A-Z, lot 19, ICL)<br>Chicken anti-GFP (cat. ab13970, lot GR3190550-6, Abcam)<br>Rabbit anti-DARPP-32 (cat. 2306S, clone 19A3, lot 7, Cell Signaling Technology)<br>Goat anti-ChAT (cat. AB144P, lot 3251012, Millipore)<br>Rabbit anti-PV (cat. PV27, lot 2014, Swant)
Validation	Mouse anti-Cre recombinase - manufacturer validated for ELISA, IC, IF, IH and WB, 182 citations.<br>Rabbit anti-FLAG (DYKDDDDK) - manufacturer validated for blotting, ELISA, and IHC, 1 citation.<br>Chicken anti-GFP - manufacturer validated for WB and ICC/IF, 2216 citations.<br>Rabbit anti-DARPP-32 - manufacturer validated for WB, IP, IHC and IF, 69 citations.<br>Goat anti-ChAT - manufacturer validated for IHC, IC, and WB, 2400 citations.<br>Rabbit anti-PV - manufacturer validated for immunoblotting and IHC, 237 citations.

Eukaryotic cell lines

Policy information about cell lines

Cell line source(s)	293T cells were obtained from Riken BRC (cat. RCB2202).
Authentication	293T cells were not authenticated.
Mycoplasma contamination	293T cells were tested negative for mycoplasma contamination at Riken BRC.

Commonly misidentified lines (See ICLAC register)	No commonly misidentified cell lines were used in this study.

Animals and other organisms

Policy information about studies involving animals; ARRIVE guidelines recommended for reporting animal research

Laboratory animals	Male C57BL/6J mice (age on the day of the first surgery, 7.9–13.0 weeks; weight, 19.9–28.6 g; SLC, Japan) were used in this study.
Wild animals	This study did not involve wild animals.
Field-collected samples	This study did not involve samples corrected from the field.
Ethics oversight	All procedures related to animal care and use were approved by the Institutional Animal Care and Use Committee of Osaka City University (approved protocol #15030) and were performed in accordance with the Guide for the Care and Use of Laboratory Animals published by the National Institutes of Health.

Note that full information on the approval of the study protocol must also be provided in the manuscript.
