# Supplementary material for: Intersectional, anterograde transsynaptic targeting of neurons receiving monosynaptic inputs from two upstream regions
Source: Commun Biol. 2022 Feb 21;5:149. doi: 10.1038/s42003-022-03096-3 (PMC8860993; doi:10.1038/s42003-022-03096-3)
Supplement: Supplementary file 5 — Editorial Request [file 42003_2022_3096_MOESM5_ESM.docx]

***To the Author****— Please review the editorial comments and requests below and confirm that changes have been made in the manuscript in the right-hand column.* ***This document*** ***must be uploaded*** *as a related manuscript file.*

Please see our [final file submission checklist](https://www.nature.com/documents/commsj-file-checklist.pdf) for information about submitting your revised documents.

| **Files and General Policies** | |
| --- | --- |
| **Main manuscript file must be in Microsoft Word or LaTeX format.**  LaTex and Tex article source files must be accompanied by the compiled PDF for reference. The bibliography must be submitted separately (as a .bib file) or contained within the .tex file. | Confirmed. |
| **Each Figure must be provided as a separate file** and must be supplied whole, with all panels included in a single document. Figures should be provided at a minimum resolution of 300 dpi at final size.  Figure files must only contain images (please also leave out labels such as “Figure 1” etc). Figure captions must instead be included within the main manuscript file, grouped together at the end of the document. | Confirmed. |
| All figures, tables, and supplementary items must be cited in the manuscript and **numbered in the order in which they appear**. | Confirmed. |
| Please check whether your manuscript contains **third-party images**, such as figures from the literature, stock photos, clip art or commercial satellite and map data. We strongly discourage the use or adaptation of previously published images, but if this is unavoidable, please request the necessary rights documentation to re-use such material from the relevant copyright holders and return this to us when you submit your revised manuscript.  An appropriate permissions statement must be present in the relative figure caption for any third-party images. | Confirmed. Our manuscript does not contain third-party images. |
| **Please check that you have not copied any text directly from published work** (even your own) without clear attribution, including one or more references. We run a plagiarism detection software and may need to request additional changes if we identify large blocks of identical text. | Confirmed. |
| An updated **editorial policy checklist** that verifies compliance with all required editorial policies must be completed and uploaded with the revised manuscript. All points on the policy checklist must be addressed; if needed, please revise your manuscript in response to these points.  <https://www.nature.com/documents/nr-editorial-policy-checklist.pdf>.  Please note that this form is a dynamic ‘smart pdf’ and must therefore be downloaded and completed in Adobe Reader. This file will not open in an internet browser. | Confirmed. |
| The **reporting summary** will be published alongside your manuscript therefore it needs to accurately represent your work. In this case, please take a closer look at the reporting summary and make sure things are completed correctly. If an item does not apply, for example human participants, I need you to check the NA box next to that item. No section should be left blank.  Also, please make sure to include your name and date at the top of the document.  If you require a new Reporting Summary form, please download it here: <https://www.nature.com/documents/nr-reporting-summary.pdf>.  Please note that this form is a dynamic ‘smart pdf’ and must therefore be downloaded and completed in Adobe Reader. This file will not open in an internet browser. | Confirmed. |
| Your paper will be accompanied by a two-sentence editor's summary when it is published on our homepage. Please approve the draft summary below or provide us with a suitably edited version.  In this paper, a method is developed to genetically label neurons that receive monosynaptic inputs from two upstream regions of the brain. This could improve the analysis of interregional integration of information in neurons. | Approved. |
| **ORCID**  *Communications Biology* is committed to improving transparency in authorship. As part of our efforts in this direction, we are now requesting that all authors identified as ‘corresponding author’ create and link their Open Researcher and Contributor Identifier (ORCID) with their account on the Manuscript Tracking System (MTS) prior to acceptance. ORCID helps the scientific community achieve unambiguous attribution of all scholarly contributions. For more information please visit <http://www.springernature.com/orcid>.  For all corresponding authors listed on the manuscript, please follow the instructions in the link below to link your ORCID to your account on our MTS before submitting the final version of the manuscript. If you do not yet have an ORCID you will be able to create one in minutes.  <https://www.springernature.com/gp/researchers/orcid/orcid-for-nature-research>  IMPORTANT: All authors identified as ‘corresponding author’ on the manuscript must follow these instructions. Non-corresponding authors do not have to link their ORCIDs but are encouraged to do so. Please note that it will not be possible to add/modify ORCIDs at proof. Thus, if they wish to have their ORCID added to the paper they must also follow the above procedure prior to acceptance.  To support ORCID's aims, we only allow a single ORCID identifier to be attached to one account. If you have any issues attaching an ORCID identifier to your MTS account, please contact the Platform Support Helpdesk at <http://platformsupport.nature.com/> | Confirmed. |
| We regularly highlight papers published in *Communications Biology* on the journal’s **Twitter** account (@CommsBio). If you would like us to mention authors, institutions, or lab groups in these tweets, please provide the relevant twitter handles in the right-hand column. | Takuma Kitanishi  @tkitanishi |
| We would welcome the submission of material for the **‘Featured Image’** section on the Communications Biology home page. Images should relate to the content of your manuscript but need not be contained within the paper. **Photographs and aesthetically interesting images are preferred; diagrams are generally not used.** Suggestions should be uploaded as a Related Manuscript file. Please provide 1200x675-pixel RGB images. You will also need to submit a completed [Image License to Publish](https://www.nature.com/documents/snl-image-ltp.docx).  Unfortunately, we cannot promise that your suggestions will be used. | We do not submit an image for Featured Image. |
| **Supplementary information** | |
| **Supplementary Information Format and referencing**   - Supplementary Figures, small Tables, and any supplementary text must be provided **in a single PDF**. Figures and their captions should be presented together.   - If you include a title page, please check that the title and author list matches the main manuscript. - **All Supplementary items must be referred to in the manuscript**, and items must be mentioned in numerical order. Please do not include general references to “Supplementary Material”; instead refer to specific items. - Additional files can be provided as **Supplementary Data** (Excel files, text files, .zip folders), **Supplementary Movies**, **Supplementary Audio**, or **Supplementary Software** (.zip folder)   Supplementary Information files will be uploaded with the published article as they are submitted with the final version of your manuscript. Any highlighting or tracked changes should be removed from the file. | Confirmed. |
| **Supplementary items must be cited in a consistent format**. Names of items in the Supplementary file(s) must match those used in the main manuscript.  **We recommend using the following naming formats:** Supplementary Figure 1, Supplementary Table 1, Supplementary Data 1, Supplementary Note 1, and Supplementary References. | Confirmed. |
| **Source data for graphs and charts:** We strongly recommend depositing these to suitable repositories (such as Figshare, Dryad, or a data type-specific repository if one exists).  Otherwise, all source data underlying the graphs and charts presented in the main figures must be uploaded as **Supplementary Data** (in Excel or text format). **Note that only the data used directly for generating the charts needs to be supplied.**  Please provide captions for each Supplementary Data item here **in the column to the right**. | All source data underlying graphs are provided as Supplementary Data 1. Caption: Source data for Figure 3k. |
| **Title Page** | |
| Please ensure that the author list provided in our manuscript tracking system matches the author list in the main manuscript. | Confirmed. |
| **Manuscript title**  Please ensure the title clearly describes the central finding of the paper. We recommend writing the title as a declarative statement of approximately 15 words or fewer.  Be sure to include any key species, protein names, or gene names to ensure optimal retrieval of the paper in database searches. | Confirmed. |
| **Abstract**  The abstract should be **accessible to non-specialists** and avoid jargon and abbreviations. Please write the abstract in the present tense.  We recommend structuring the abstract as follows:  Opening statement explaining why this area of research is important. A sentence explaining the gap in knowledge that your research will address. **Here we show** (or an equivalent phrase), and then the major results and conclusions of the paper. Final sentence indicating any broader impacts and how this research will be used in the future. | Confirmed. |
| **Main text** | |
| **Format of the main text**  Please ensure your manuscript includes the following sections, presented in this order:   1. “**Introduction”**: The background and rationale for the work. The final paragraph should be a brief summary of the major results and conclusions. The results of the current study must only be discussed in this final paragraph. The Introduction should contain no references to figures or tables. Do not include subheadings. 2. “**Results” or “Results and Discussion”**. This should be split into subheaded sections; we recommend 1 subheading per main figure or table. Figures should not be embedded in the text but submitted separately.    1. Do not use more than 1 layer of subheadings.    2. A “Conclusions” paragraph can be included **only if the results and discussion are combined into a single section.** 3. “**Discussion” (optional)**, without subheadings. 4. **Methods**, which should be split into subheaded sections. Do not use more than 1 layer of subheadings.   **To improve readability**, we recommend that the main text (Introduction, Results and Discussion) be limited to approximately 5000 words or fewer. | Confirmed. |
| The discussion should consist of an extended analysis of the results and their comparison to the literature rather than a short summary or conclusion. The editors recommend the following edits:  **Please add a summary/concluding paragraph.** | Confirmed. |
| **Statistical reporting**  Wherever statistics have been derived (e.g. error bars, box plots, statistical significance) the legend needs to provide and define the n number (i.e. the sample size used to derive statistics) as a precise value (not a range), using the wording “n=X biologically independent samples/animals/independent experiments” etc. as applicable. | Confirmed. |
| **Statistical representation**  Statistics such as error bars cannot be derived from n < 3 and must be removed from all such cases. We strongly discourage deriving statistics from technical replicates, and they should be removed from all such cases, unless there is a clear scientific justification for why providing this information is important. Conflating technical and biological variability, e.g. by pooling technically replicate samples across independent experiments is strongly discouraged. | Confirmed. |
| **Please include exact p-values where possible.** We ask that you also include the name of the statistical test and the estimated effect size. If applicable, please also include the confidence interval. | Confirmed. |
| Avoid the use of the word “**significant**” unless referring the results of a statistical test. | Confirmed. |
| **Please check that all gene and mRNA names are in italics**. Protein names should not be in italics. Please confirm that only official gene/protein symbols are used and that species names are in italics. | Confirmed. |
| Language such as “new”, “novel”, “for the first time”, “unprecedented”, etc, should be avoided, or qualified with “**to the best of our knowledge**” or similar, because it often leads to unproductive controversy. **Novelty should be made clear from the context.** | Confirmed. |
| All data that support the conclusions drawn must be presented in the manuscript unless they are published elsewhere. **We do not allow statements of “data not shown”.** | Confirmed. |
| Our style does not allow for the use of bold or italics to convey emphasis. Please remove such usage from your manuscript. | Confirmed. |
| Use of speech marks around words or phrases should be avoided; if a phrase is non-standard, please explain the meaning instead; otherwise they are usually unnecessary. | Confirmed. |
| **Display items** | |
| **Figure captions/legends**  Figures must have a title that will appear above the Figure **and** a legend that will appear below the Figure (see e.g. <https://www.nature.com/articles/s42003-020-1059-1/figures/1>)  The Figure title must describe the Figure as a whole and must not contain reference to specific figure panels.  The Figure legend must refer to and describe **all panels**. Abbreviations, symbols, colors, and shading present in the Figure must be defined. Please write out the symbols/colors in words (blue circles, red dashed line, etc.) within these definitions.  **All figure panels must be labelled using lower case letters. Please refrain from referring to sections of figures as top/bottom/left/right/, etc.** | Confirmed. |
| **Axis and panel labels will be published as received.** We recommend using a sans-serif font such as Arial or Helvetica. | Confirmed. |
| **Data presentation in bar graphs and line graphs**  For all graphs depicting a single point value (e.g., mean) with error bars, **you must add individual data points or convert the graph to a boxplot or dot-plot**. You may wish to refer to [this blog post](https://ecrlife420999811.wordpress.com/2018/07/10/beyond-bar-graphs-free-tools-and-resources-for-creating-more-transparent-figures-for-small-datasets/) about representing data distribution in plots (particularly for small datasets). We strongly encourage the same for plots with multiple time courses depicted.  See the [June 24, 2019 CommsBio editorial](https://www.nature.com/articles/s42003-019-0489-0) for more details about this policy. Example plots are shown here:  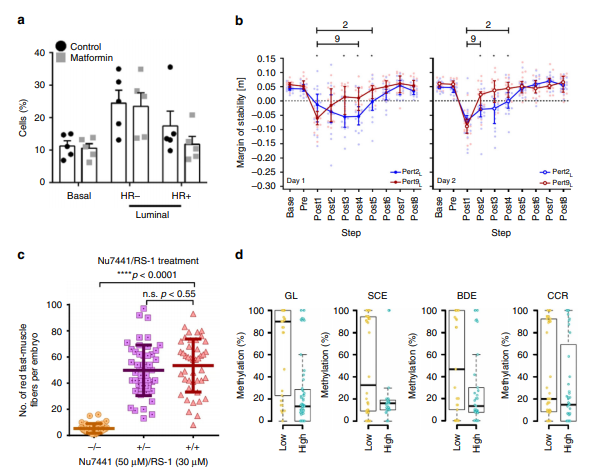  *Examples of plots showing data distribution. Figure 2 from the editorial linked to above.*  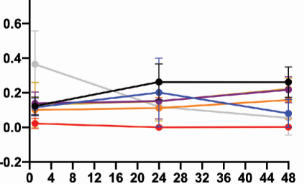  *Multiple line plot: Before*  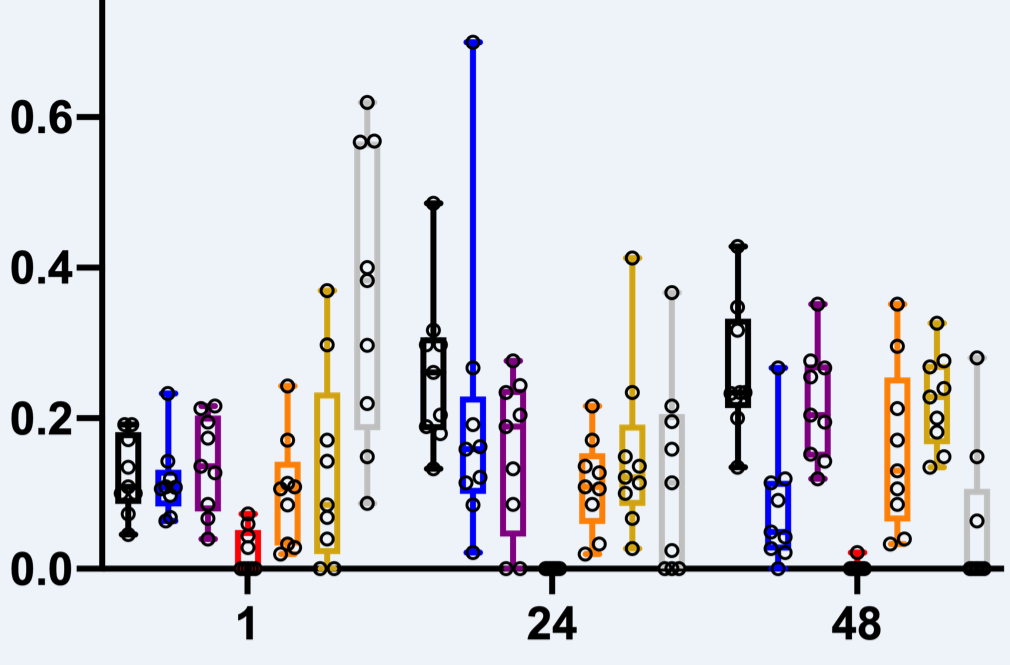  *Multiple line plot: After (converted to box plots)* | Individual data points were added to Figure 3k. |
| **When choosing a color scheme** please consider how it will display in black and white (if printed), and to users with color blindness. Please consider distinguishing data series using line patterns rather than colors, or using optimized color palettes such as those found at <https://www.nature.com/articles/nmeth.1618>.  The use of colored axes and labels should be avoided.  Please avoid the use of red/green color contrasts, as these may be difficult to interpret for colorblind readers. | Confirmed. |
| Please define the **error bars** in each Figure and Supplementary Figure where they are used. One statement at the end of each Figure caption is sufficient if the error bars are equivalent throughout the Figure. | Confirmed. |
| **Microscopy images and photographs** in each Figure and Supplementary Figure must be accompanied by scale bars, and these must be defined. | Confirmed. |
| Please pay close attention to our [Digital Image Integrity Guidelines](https://www.nature.com/nature-research/editorial-policies/image-integrity). Also ensure that you retain unprocessed data and metadata files after publication, ideally archiving data in perpetuity, as these may be requested during the peer review and production process or after publication if any issues arise. | Confirmed. |
| **Methods** | |
| Please ensure that all information present in the Reporting Summary is also in the manuscript. This information is usually most appropriate in the Methods section. | Confirmed. |
| **We allow unlimited space for Methods.** The Methods must contain sufficient detail such that the work could be repeated. It is preferable that all key methods be included in the main manuscript, rather than in the Supplementary Information.  **Please avoid use of “as described previously”** or similar, and instead detail the specific methods used with appropriate attribution. | Confirmed. |
| **The Methods should include a separate section titled “Statistics and Reproducibility”** with general information on how the statistical analyses of the data were conducted, and general information on the reproducibility of experiments, including the sample sizes and number of replicates and how replicates were defined. | Confirmed. |
| If applicable, all **oligo sequences, concentrations of antibodies, and sources of cell lines** must be included in the Methods (these can also be provided in a main Table and cited in the Methods). | Confirmed. |
| If the work involves any **cancer cell lines** that are listed in the database of commonly misidentified cell lines, ICLAC (<http://iclac.org/databases/cross-contaminations>), please provide justification for their use in the methods section. Please also state from where the lines were obtained; whether they were tested for mycoplasma contamination; and whether they were authenticated, and if so, by which method. | Confirmed. |
| For studies using **live vertebrates**, a statement affirming that you have complied with all relevant ethical regulations for animal testing and research is necessary. A statement explicitly confirming if the study received ethical approval, including the name of the board and institution that approved the study protocol is also required. The species, strain, sex and age of animals should be included. | Confirmed. |
| **Data Policies** | |
| The **Data Availability** **statement** must include:   - Access details for deposited data, including repository name and unique data ID. - How source data can be obtained. - A statement that all other data are available from the corresponding author (or other sources, as applicable) on reasonable request. **Note that ‘available upon request’ is only appropriate if immediate data access has not been mandated by our policies or by the editors.**   See here for more information about formatting your Data Availability Statement: <http://www.springernature.com/gp/authors/research-data-policy/data-availability-statements/12330880> | Confirmed. |
| **Mandatory deposition** of raw and processed data is required for:   - All sequencing data (DNA, RNA, protein) - Novel human genetic polymorphisms (e.g., [dbSNP](https://www.ncbi.nlm.nih.gov/snp/)) - Linked genotype and phenotype data (e.g., [dbGaP](https://www.ncbi.nlm.nih.gov/gap/) for human data) - GWAS summary statistics or polygenic risk scores - Novel macromolecular structure - Gene expression microarray data (must be MIAME compliant) - Crystallographic data for small molecules - Mass spectrometry-based proteomics data   For more information on mandatory data deposition policies at the Nature Portfolio, please visit <http://www.nature.com/authors/policies/availability.html#data>  For an up-to-date list of approved repositories for each mandatory data type, please visit <https://www.springernature.com/gp/authors/research-data-policy/repositories/12327124>.  **Accession code(s) for deposited data must be provided in the Data Availability statement in the final version of the paper.** Failure to do so will delay publication. Please ensure data are available prior to publication. | Confirmed. A newly constructed plasmid, pAAV-hSyn-Flpo-3×FLAG, and its sequence data was deposited to Addgene (no. 173047). |
| *Communications Biology* has a strong preference for all data to be deposited in an approved repository. In some cases, data deposition may be required by the editor.  **We recommend the following data repositories:**   - [GenBank](https://www.ncbi.nlm.nih.gov/genbank/) (all DNA sequence data) - [NHGRI-EBI GWAS Catalog](https://www.ebi.ac.uk/gwas/) (GWAS summary statistics) - [PGS Catalog](https://www.pgscatalog.org/) (polygenic risk scores) - [Gene Expression Omnibus](https://www.ncbi.nlm.nih.gov/geo/) (Microarray or RNA sequencing data) - [Sequence Read Archive](https://www.ncbi.nlm.nih.gov/sra) (WGS or WES data) - [Protein Data Bank](https://www.wwpdb.org/) (protein structural data) - [OSF](https://osf.io/) (neuroimaging raw data and EEG/EMG/MEG raw data) - [Neurovault](https://neurovault.org/) (unthresholded statistical maps, parcellations, and atlases produced by MRI and PET studies) - [Image Data Resource](https://idr.openmicroscopy.org/about/) (microscopy data) - [PRIDE](https://www.ebi.ac.uk/pride/) (proteomics data)   Data types without a specific repository can be deposited in a generalist repository, such as [figshare](https://figshare.com/) or [Dryad](https://datadryad.org/stash).  For an up-to-date list of approved repositories, please visit <https://www.springernature.com/gp/authors/research-data-policy/repositories/12327124>. | Confirmed. |
| **Data citation**  Please cite datasets stored in external repositories **in the main reference list**.  For previously published datasets, we ask authors to cite both the related research articles and the datasets themselves.  For more information on how to cite datasets in submitted manuscripts, please see our [data availability statements and data citations policy](https://www.nature.com/documents/nr-data-availability-statements-data-citations.pdf). | Confirmed. The present work did not use datasets in external repositories. |
| Please deposit your **newly generated plasmids** in a community repository (eg, Addgene). Include the ID numbers in the Data Availability statement. | A newly constructed plasmid, pAAV-hSyn-Flpo-3×FLAG, was deposited to Addgene (no. 173047). This information was provided in the Data Availability statement. |
| **Data publishing**  You may also be interested in publishing a Data Descriptor (<https://www.nature.com/sdata/>) or Data Note (<https://bmcresnotes.biomedcentral.com/>) to improve the usability of your data set. | Confirmed. |
| **End Notes** | |
| Please check that your bibliography complies with the following:   - Your bibliography should start with the heading “References”. The references must be numbered in the order of appearance in the text, then tables, then figures. - Any in-text citations to references (e.g. "Gupta et al. show...") should be followed by their corresponding reference citation number from the reference list. - Manuscript citations must include journal title, article title, volume number, page or article number or DOI, and year of publication. - No publication can be present more than once in the reference list. - No footnotes are permitted in the references or elsewhere. Text should be incorporated into the main text, the Methods section, or the Supplementary Information instead. - Websites should only be listed in the references if they are in common use or curated. - Where possible, preprints in the reference list should be updated with details of the published, peer-reviewed paper. - Citations should be formatted in the text using superscript numbers. | Confirmed. |
| Please check that your '**Author Contributions'** section individually lists the specific contribution of each author to the work. Each author must be referred to by name or initials. Where multiple authors possess identical initials, they must be clearly disambiguated from one another.  See our author contributions policy for further information: <https://www.nature.com/nature-research/editorial-policies/authorship#author-contribution-statements> | Confirmed. |
| No separate funding section is permitted. Please include your funding information in Acknowledgements. | Confirmed. |
