# Supplementary material for: Intersectional, anterograde transsynaptic targeting of neurons receiving monosynaptic inputs from two upstream regions
Source: Commun Biol. 2022 Feb 21;5:149. doi: 10.1038/s42003-022-03096-3 (PMC8860993; doi:10.1038/s42003-022-03096-3)
Supplement: Supplementary file 6 — Style and formatting checklist [file 42003_2022_3096_MOESM6_ESM.pdf]

Style and formatting checklist

Primary Research Articles

communications biology

Manuscripts must be formatted as below at the point of acceptance. Manuscripts do not need to be formatted at submission or during revision.

Main manuscript		
Author list	The author list provided in your article must match that provided on our manuscript tracking system.	☒
Author list	Job titles must not be included in the affiliations or author list.	☒
Author list	Affiliations must be numbered in the order of their first appearance in the author list.	☒
Author list	Each affiliation must contain only one address.	☒
Author list	Author affiliations must include institution, city and country.	☒
Author list	Author tagging statements are limited to the following two options: "These authors contributed equally" and "These authors jointly supervised this work", with no more than one of each tag permitted.<br>Where relevant, 'present address' must be provided separately as the final affiliation.	☒
Author list	At least one corresponding author must be designated, with a limit of four.	☒
Author list	An e-mail address must be provided for each corresponding author, and only one e-mail address is permitted per corresponding author.	☒
Author list	For each corresponding author, and for any author who wishes for their ORCID to be included in the final article, an ORCID should be included in their author profile in our manuscript tracking system prior to formal acceptance. ORCIDs cannot be added at the proof stage.	☒
Author list	No more than six authors can be designated as equally contributing.	☒
Author list	No more than six authors can be designated as jointly supervising.	☒
Abstract	An abstract must be provided and must begin with the heading 'Abstract'. The abstract must contain background information and a summary of the present work. It must not contain references.	☒
Abstract	Graphical abstracts are not supported and should not be supplied.	☒
Introduction	An introduction must be provided and must begin with the heading 'Introduction'. The introduction should include the background and rationale for the work. The final paragraph should be a brief summary of the major results and conclusions.	☒
Language and style	Text must not be copied directly from published work (even your own) without clear attribution, including one or more references.	☒
Language and style	Acronyms should be defined upon first use. Non-standard two letter abbreviations should be avoided. Acronyms and abbreviations should be used only if they appear three or more times in the text. No list or table of abbreviations/definitions is permitted.	☒
Language and style	Mathematical terms should conform to the following guidelines:<br>➢ Scalar variables (e.g. x, V, χ) should be typeset in italics.<br>➢ Multi-letter variables should be formatted in Roman.<br>➢ Constants (e.g. ħ, G, c) should be typeset in italics (the only exceptions being e, i, π, which should be typeset in Roman) and vectors (such as r, the wavevector k, or the magnetic field vector B) should be typeset in bold without italics.<br>➢ In contrast, subscripts and superscripts should only be italicised if they too are variables or constants. Those that are labels (such as the 'c' in the critical temperature, T_c, the 'F' in the Fermi energy, E_F, or the 'crit' in the critical current, I_{crit}) should be typeset in Roman.<br>➢ To avoid doubt, unit dimensions should be expressed using negative integers (e.g.	☒

	kg m^{-1} s^{-2}, not kg/ms^2) or the word 'per'.	
Results	A section titled ‘Results’ or ‘Results and Discussion’ must be provided. This section must include no more than two levels of subheadings.	☒
Results	All data that support the conclusions drawn must be presented in the manuscript unless they are published elsewhere. We do not allow statements of “data not shown”.	☒
Results	References to "personal communication" in the text require written permission from cited person.	☒
Results	Supplementary items must be cited in a consistent format throughout the manuscript, and in line with the labels used in the Supplementary Information. We recommend using the following formats: Supplementary Figure 1, Supplementary Table 1, Supplementary Methods, Supplementary Note 1, Supplementary Discussion, Supplementary References, Supplementary Movie 1, Supplementary Audio 1, Supplementary Data 1 and Supplementary Software 1.	☒
Results	All Supplementary items should be cited in the main manuscript.	☒
Results	Equations must be supplied in an editable format, and not as images.	☒
Results	Where numbered equations are provided, they must appear in sequential order.	☒
Display items	Schemes are not permitted and must be labelled as Figures instead.	☒
Display items	Chemical reactions must be labelled as Equations if displayed as text, or as Figures if displayed as images.	☒
Display items	Figure captions must start with a brief title that describes the Figure as a whole and does not contain reference to specific figure panels.	☒
Display items	Figure captions must also have a legend that defines each Figure panel individually. Figure panels must be labelled with lower case letters, and all panels must be defined in order in the legend.	☒
Display items	For all graphs depicting a single point value (e.g., mean) with error bars, please add individual data points or convert the graph to a box-plot or dot-plot. This applies to bar graphs and line graphs	☒
Display items	Abbreviations, symbols, colours and shading must be defined, even if previously defined in the main text.	☒
Display items	Where error bars are used, they must also be defined. Statistics such as error bars cannot be derived from n<3, and must be removed from all such cases. We strongly discourage deriving statistics from technical replicates, and they should be removed from all such cases, unless there is a clear scientific justification for why providing this information is important. Conflating technical and biological variability, e.g. by pooling technically replicate samples across independent experiments is strongly discouraged.	☒
Display items	Microscopy images and photographs must be accompanied by scale bars. Please avoid the use of red/green color contrasts, as these may be difficult to interpret for colorblind readers.	☒
Display items	Tables must have a caption. Abbreviations and symbols/variables listed in Tables must be defined in the Table caption.	☒
Display items	Data in Tables must be free from bold/italic formatting unless this has been clearly defined in the footnote. Tables must only contain black and white text, with no colours or shading.	☒
Display items	Where Tables contain images, each image should appear in its own cell in the absence of any text.	☒
Display items	All Figures and Tables must be cited in the text in numerical order.	☒
Methods	A Methods section must be provided (except for purely theoretical work).	☒
Methods	A life sciences Reporting checklist should be provided. https://www.nature.com/documents/nr-reporting-summary.zip	☒
Methods	An Editorial Policy checklist should be provided. https://www.nature.com/documents/nr-editorial-policy-checklist.zip	☒

Methods	The information present in the Nature Research Reporting Summary should also be present in the manuscript.	☒
Methods	The Methods and Supplementary Methods must together include sufficient detail such that the work could be reproduced.	☒
Methods	For studies using live vertebrates, a statement affirming that you have complied with all relevant ethical regulations for animal testing and research is necessary. A statement explicitly confirming if the study received ethical approval, including the name of the board and institution that approved the study protocol is also required. The species, strain, sex and age of animals should be included.	☒
Methods	Please ensure that you have included any oligo sequences, sources and concentration of antibodies and sources of all cell lines, if applicable.	☒
Methods	If the work involves any cancer cell lines that are listed in the database of commonly misidentified cell lines, ICLAC (http://iclac.org/databases/cross-contaminations), please provide justification for their use in the methods section. Please also state from where the lines were obtained; whether they were tested for mycoplasma contamination; and whether they were authenticated, and if so, by which method.	☒
Methods	Where human participants are involved, confirmation that all relevant ethical regulations were followed is needed, and that informed consent was obtained. This must be stated in the Methods section, including the name of the board and institution that approved the study protocol.	☒
Methods	The Methods should include a separate section titled “Statistics and Reproducibility” with general information on how the statistical analyses of the data were conducted, and general information on the reproducibility of experiments, including the sample sizes and number of replicates and how replicates were defined.	☒
Methods	Details of geological samples and palaeontological specimens that include clear provenance information are needed. Information regarding the requisite permission obtained is required. Palaeontological and type specimens should be deposited in a recognised museum or collection.	☒
Data	A “Data Availability” section must be provided. The paper must conform to our requirements on mandatory data deposition, see https://www.springernature.com/gp/authors/research-data-policy.	☒
Data	For novel protein structures, a stereo image of a portion of the electron density map (including contour level and type of map) must be provided for crystallographic structures, or the superimposed lowest energy structures (>10) for NMR structures. Structural data must be deposited in the relevant publicly accessible database, and accession codes must be provided in the data availability section.	☒
Data	Extended data items are not permitted and must be placed in the Supplementary Information instead.	☒
Data	All source data underlying the graphs and charts in the main figures must be uploaded either as Supplementary Data or deposited in a generalist repository (such as figshare or Dryad) and cited in the main manuscript.	☒
Data	Where portions of blots and gels have been presented in the main paper, the full blot or gel should be included in the Supplementary Information file.	☒
Data	Please use the Nature templates for NMR, cryo-EM, and X-ray refinement statistics for newly reported macromolecular structures (see https://www.nature.com/commsbio/submit/submission-guidelines#characterisation). These should be presented as Tables in the main manuscript file.	☒
Code	For all studies developing new software or using custom code that is deemed central to the conclusions, a Code Availability section must be included indicating whether and how the code can be accessed, including any restrictions to access. This section should also include information on the versions of any software used, if relevant, and any specific variables or parameters used to generate, test, or process the current dataset.	☒
References	References must be listed by number and should be given in the order of appearance in the text, then tables, figures and finally boxes.	☒
References	Each reference must contain only one item; compound references are not permitted.	☒

References	Manuscript citations must include journal title; article title; volume number; start and end page or article number or DOI; and year of publication, in this order.	☒
References	No publication can be present more than once in the reference list.	☒
References	No footnotes are permitted in the references or elsewhere. Text must instead be incorporated into the main text, the Methods section, or the Supplementary Information.	☒
References	Only articles that have been published or accepted by a named publication or recognised preprint server can be listed in the References. Other persistent and recognised sources such as books and monographs are also permitted. Websites should only be cited if they are in common use or curated.	☒
Acknowledgements	Any acknowledgements must be relevant, appropriate and kept to a minimum. No separate funding section is permitted. Funding information should be included in the Acknowledgements.	☒
Author contributions	An author contributions statement that individually lists the specific contributions of each author to the work must be provided. All authors must be referred to by name or initials in this statement. All authors must be clearly disambiguated from one another.	☒
Competing interests	A competing interests statement that includes both financial and non-financial interests must be provided. All authors must be accounted for within the statement, e.g. with use of 'all other authors declare no competing interests' where relevant.	☒

Supplementary Information	
We do not edit Supplementary Information files; they will be uploaded with the published article as they are submitted with the final version of your manuscript. Any tracked changes should be removed from the file.	☒
Any information provided on the cover/title page of the Supplementary Information must match the title and author list in the main manuscript.	☒
We recommend labelling and citing Supplementary items using the following formats: Supplementary Figure 1, Supplementary Table 1, Supplementary Methods, Supplementary Note 1, Supplementary Discussion, and Supplementary References. All supplementary items must be cited in the main article.	☒
Each Supplementary Figure requires a caption. These should be presented below the Figure and should refer to all panels.	☒
Error bars in Supplementary Figures must be defined.	☒
Where data are presented in Supplementary Information, large datasets must be supplied as Supplementary Data files, whereas smaller Tables must be supplied as Supplementary Tables.	☒
Where Supplementary Movie/Audio/Data/Software files are provided, each file should be labelled as Supplementary Movie/Audio/Data/Software 1, etc., and legends for these should be given in the cover letter (not in the Supplementary Information file).<br><br>All Supplementary Movie/Audio/Data/Software must be cited in the main manuscript.	☒
