# Supplementary material for: Intersectional, anterograde transsynaptic targeting of neurons receiving monosynaptic inputs from two upstream regions
Source: Commun Biol. 2022 Feb 21;5:149. doi: 10.1038/s42003-022-03096-3 (PMC8860993; doi:10.1038/s42003-022-03096-3)
Supplement: Supplementary file 8 — Revised manuscript submission file checklist [file 42003_2022_3096_MOESM8_ESM.pdf]

Revised manuscript submission file checklist

communications biology

Should you have any questions regarding this checklist, please contact us at commsbio@nature.com
This checklist is for your own use and the formatting guidelines are not mandatory at this stage. You do not need to resubmit this checklist.

Formatting guidelines & Policy		
**Item**	**Notes**	**Completed**
Title	Titles should be approximately 15 words or fewer.	☑
Abstract	As a guide, we recommend no more than 150 words. References are not allowed.	☑
Main text	As a guide, we recommend that the Introduction, Results, and Discussion should be no more than 5,000 words combined. See our submission guidelines for further details.	☑
Methods	We allow unlimited space for methods. Any relevant protocols should be described within the text to ensure reproducibility. Details can be found here.	☑
Figures and tables	• Up to 10 display items (figures + tables) are allowed in the main text.<br>• Convert all bar graphs to box-and-whisker or dot-plot format to show data distribution.<br>• Figure legends should include sample sizes and statistics where applicable.<br>• Tables must be in Word-editable format.<br>• Please refer to the Nature Research image integrity policies.	☑
Supplementary materials	• The Supplementary Information should be organised into a single PDF (excluding any non-flat files). Supplementary figure legends should be placed directly beneath the respective figure.<br>• Large data tables should be uploaded separately in Excel or similar format as 'Supplementary Data'.	☑
Statistics	The Methods should include a separate section titled "Statistics and Reproducibility" with general information on how the statistical analyses of the data were conducted, and general information on the reproducibility of experiments, including the sample sizes and number of replicates and how replicates were defined.<br>Further policy details regarding statistics can be found here.	☑
Data	A Data Availability statement is mandatory. Some data types require deposition into an approved repository. For more information on mandatory data deposition policies at Nature Research, please visit https://www.nature.com/nature-research/editorial-policies/reporting-standards#data.	☑
Data sources	We strongly encourage authors to deposit all new data associated with the paper in a persistent repository where they can be freely and enduringly accessed. We recommend submitting the data to discipline-specific, community-recognized repositories, where possible and a list of recommended repositories is provided here.<br>If a community resource is unavailable, data can be submitted to generalist repositories such as figshare or Dryad. Please include a link for reviewers to view any deposited data in the manuscript and indicate in the cover letter where you have deposited your data.<br>If you need help complying with this policy, or need help depositing and curating your research data (including raw and processed data, text, video, audio and images) you should consider:<br>• Contacting Springer Nature's free Research Data Helpdesk for advice	☑

	• Finding a suitable data repository for your data<br>• Uploading your data to Springer Nature's Research Data Support service*<br>*Please note there are fees for using Springer Nature's Research Data Support service.	☑
Source data for figures	All source data underlying the graphs and charts presented in the main figures must be made available as Supplementary Data (in Excel or text format) or via a generalist repository (eg, Figshare or Dryad).	☑
Ethics	• Include a Competing Interests statement<br>• Check that your manuscript complies with our ethics and biosecurity policies.	☑
Third party rights	Please check whether your manuscript contains third-party images, such as figures from the literature, stock photos, clip art or commercial satellite and map data. We strongly discourage the use or adaptation of previously published images, but if unavoidable please request the necessary rights documentation and return this to us when you submit your revised manuscript.	☑
Other notes	• We do not allow 'data not shown' statements.<br>• Please avoid claims of novelty.	☑
Structural studies	Use the Nature templates for NMR, cryo-EM, and X-ray refinement statistics for newly reported macromolecular structures. Tables should be in the main text.	☑
Studies reporting new species	Please obtain LSID(s) and add the following section to the Methods:<br>Nomenclatural Acts.<br>This published work and the nomenclatural acts it contains have been registered in ZooBank, the proposed online registration system for the International Code of Zoological Nomenclature (ICZN). The ZooBank LSIDs (Life Science Identifiers) can be resolved and the associated information viewed through any standard web browser by appending the LSID to the prefix ''http://zoobank.org/''. The LSIDs for this publication are: xxxxxxxxxx; yyyyyyyyyy.	☑
Studies reporting palaeontological data	Details of geological samples and palaeontological specimens that include clear provenance information are needed. Information regarding the requisite permission obtained is required. Palaeontological and type specimens should be deposited in a recognised museum or collection.	☑
Cancer cell lines	If the work involves any cancer cell lines that are listed in the database of commonly misidentified cell lines, ICLAC (http://iclac.org/databases/cross-contaminations), please provide justification for their use in the methods section. Please also state from where the lines were obtained; whether they were tested for mycoplasma contamination; and whether they were authenticated, and if so, by which method.	☑

Response to reviewers		
**Item**	**Notes**	**Completed**
Referee comments	Be sure to include all referee comments without editing or omitting any text. We recommend that you number comments if this was not already done.	☑
Replies	Ensure each reply is presented together with the relevant referee comment. We suggest formatting the reply within a table, with referee comments in one column and responses in another (this is not mandatory).	☑
	Please indicate all changes to the manuscript by line number. We also recommend including the revised text within the reply, unless it is very long.	☑
	Any updated figures should be shown within the reply document. Please specify what changes were made.	☑

Submission Files			
**Item**	**Permissible file format**	**Notes**	**Completed**
Cover letter	.doc, .docx, .pdf	Outline any major changes to the manuscript.	☑
Point-by-point response to reviewers	.doc, .docx, .pdf	See 'Response to reviewers' table above	☑
Article File	.doc, .docx, pdf	• Only one file permitted. At this stage, you may include the figures and tables within the manuscript file in their appropriate location within the text.<br>• All changes should be indicated in the manuscript file, by highlighting or by using Track Changes.<br>• Please upload the article as an 'Article' file. Do not use the 'Revised manuscript – marked up' file format.	☑
Supplementary Information	.txt, .gif, .html, .doc, .jpg, .swf, .mov, .xlsx, .pdf, .ppt, .wav, .csv, .zip	Can be uploaded separately or included within the Article file. Supplementary datasets should be provided as separate .xlsx files and uploaded as 'Supplementary Data' files.	☑
Reporting summary	.pdf	An updated version of the reporting summary. You can download the reporting summary here: https://www.nature.com/documents/nr-reporting-summary.zip	☑
Editorial policy checklist	.pdf	An updated version of the Editorial Policy Checklist can be downloaded here: https://www.nature.com/documents/nr-editorial-policy-checklist.zip	☑
